# Comparative wound healing of intestinal anastomosis: high-frequency electric welding vs suture in a rabbit model

**DOI:** 10.1097/JS9.0000000000004571

**Published:** 2026-01-12

**Authors:** Caihui Zhu, Xiaonan Tao, Yuyan Na, Xiujun Cheng, Hui Zhao, Jian Qiu, Xiaodong Gu, Jianbin Xiang, Kefu Liu

**Affiliations:** a College of Intelligent Robotics and Advanced Manufacturing, Fudan University, Shanghai, China; b Department of Sports Medicine, The Second Affiliated Hospital of Inner Mongolia Medical University, Hohhot, Inner Mongolia Autonomous Region, China; c Department of Dermatology, Huashan Hospital, Fudan University, Shanghai, China; d Department of General Surgery, Huashan Hospital, Fudan University, Shanghai, China

**Keywords:** HFEW, intestinal anastomosis, in vivo, side-to-side, suture, wound healing

## Abstract

**Background:**

This study aimed to comparatively evaluate the tissue healing characteristics of High-Frequency Electric Welding (HFEW) versus conventional suture techniques in intestinal side-to-side anastomosis using an animal model.

**Materials and methods:**

Intestinal side-to-side anastomoses were performed in New Zealand White rabbits using HFEW and hand-sewn techniques. Intraoperative assessment focused on procedure duration, hemostasis, and immediate anastomotic strength. Postoperative monitoring tracked vital signs, physiological parameters, healing progression (assessed via burst pressure), histopathological features, and inflammatory cell infiltration.

**Results:**

HFEW application reduced surgical duration and postoperative bleeding. Healing progression was comparable between HFEW and Suture groups. At 1 week postoperatively, vital signs and tissue strength normalized in experimental animals, with necrotic tissue replaced by regenerated tissue; Suture group specimens exhibited elevated inflammatory cell infiltration. By week 2, tissue strength further improved, inflammation subsided, and tissue morphology advanced. At 4 weeks, pathological structures in both HFEW and Suture groups closely resembled normal tissue. Compared with sutures, HFEW has advantages in terms of operating time and blood loss. During the postoperative healing phase, HFEW can effectively reduce the inflammatory response and accelerate healing.

**Conclusions:**

Postoperative healing with HFEW-based intestinal repair demonstrates comparability to manual suturing, while exhibiting reduced complication rates – offering residue-free foreign body anastomosis solution for laparoscopic surgery.

## Introduction

Intestinal reconstruction is a common surgical procedure typically employed to reconnect two separated intestinal segments after an intestinal resection such as intestinal tumors, inflammatory bowel diseases^[1]^, intestinal perforation, or abscesses^[2]^. Currently, there are two primary methods for intestinal reconstruction: hand sewn and stapler^[3–5]^. Hand sewn requires high surgical skills^[4]^.While stapler comes with the issue of foreign body residue, which can lead to complications at the anastomotic site (AS)^[4,6–9]^. Advancements in minimally invasive surgery urgently demand innovative anastomotic techniques to overcome limitations of manual suturing and stapler anastomosis.

**HIGHLIGHTS**

- This study compared the postoperative healing mechanisms of HFEW technology and suture technology through *in vivo* experiments on New Zealand rabbits.
- The results showed that HFEW has a high level of security and reliability, with less bleeding, faster maneuvering, and lower inflammatory response.
- This study strengthens our understanding of anastomoses without foreign body residue and has far-reaching implications for the clinical application of HFEW.

High-Frequency Electric Welding (HFEW) is an advanced tissue fusion technique^[10]^. The core principle involves the application of high-frequency electrical currents to the tissue, leading to a controllable and gradual temperature rise. This precise thermal impact induces collagen denaturation of tissue, promoting tissue adhesion^[11,12]^. Ukrainian research in the field of HFEW has proved that HFEW is expected to replace the traditional anastomosis methods and thus become the third generation of anastomosis devices^[13,14]^. However, research on HFEW is currently limited to the *in vitro* phase^[15]^, and its safety and healing mechanisms remain unclear.

After treatment with HFEW, AS undergoes a major transformation in the form of thinning, hardening and loss of bioactivity of the tissue^[16,17]^. While the tissue at the anastomotic junction has undergone necrosis, it remains essential and cannot be excised due to its crucial role in maintaining the integrity of the anastomotic connection. Previous studies have focused on the effects of physical parameters (e.g., compression pressure, power, and time) on anastomotic strength, and little research has been done on the healing characteristics^[18–22]^. Moreover, *in vitro* experiments are constrained in their capacity, merely capable of elucidating the adhesive mechanisms of HFEW^[16]^, while remaining insufficient to expound upon the mechanisms underlying anastomotic regeneration and repair. Three critical issues must be addressed in *in vivo* experiments. First, it is essential to determine whether the necrotic tissue, which has a certain level of anastomotic strength, can effectively and temporarily occlude tissues within the organism. Second, an investigation is needed to assess whether the necrotic tissue has the potential to regenerate and reshape within the organism, ultimately transforming into a durable, permanent closure tissue. Third, potential advantages brought by HFEW should be further clarified in the healing process compared to conventional suturing techniques. To address the above issues, this study proposes to design an *in vivo* experiment in New Zealand to compare the postoperative healing characteristics of HFEW and traditional suturing techniques. This study will help gastrointestinal surgeons gain a deeper understanding of HFEW, allowing them to better appreciate its potential benefits in surgical procedures. Additionally, it provides crucial experimental support for medical device experts in the field of electrosurgery, enabling them to innovate and enhance their designs based on actual surgical needs when developing new medical devices. This work has been reported in line with TITAN guidelines^[23]^.

To characterize the comparative *in vivo* healing profiles of HFEW and manual suturing, we conducted cecal closure procedures in New Zealand rabbits. This investigation systematically assessed operative efficiency (duration, intraoperative bleeding), immediate anastomotic strength, procedural safety/reliability, and longitudinal postoperative healing progression with inflammatory response monitoring.

## Materials and methods

### Ethical approval

Rabbits (*n* = 57) were sourced from Shanghai Jiao Tong University’s School of Agriculture and underwent a thorough 10-day acclimatization period before experiments. All procedures were approved by the Ethics Committee of Shanghai Jiao Tong University (Permit Number 20200701). The work has been reported in accordance with the ARRIVE guidelines (Animals in Research: Reporting *In Vivo* Experiments)^[24]^.

### Anastomotic device

**Figure 1.**
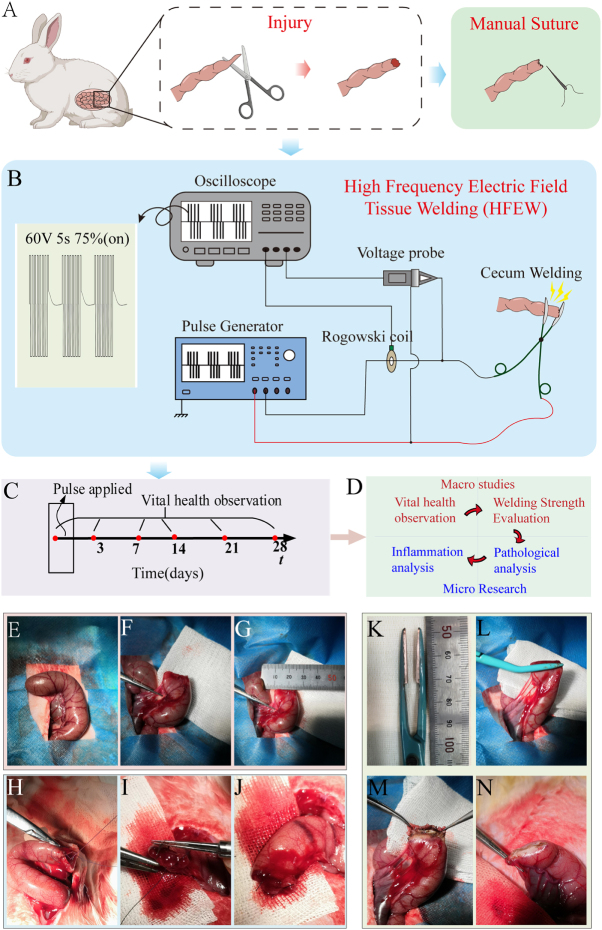
Experimental setups and surgical procedures. (A) Schematic diagram of needle and thread suture closure of the end of the cecum in New Zealand rabbits. (B) Schematic diagram of the high-frequency electric field tissue welding technique (HFEW). (C and D) Observations of postoperative vital signs in experimental animals. (E–J) Surgical procedure for hand-stitching closure of the end of the cecum. (K–N) Surgical procedure for the closure of the end of the cecum by applying the HFEW method.

*In vivo* animal experiments were divided into control (*n* = 3), Suture (*n* = 27), and HFEW(*n* = 27) groups (Supplemental Digital Content Table S1, available at: http://links.lww.com/JS9/G555). All the experimental animals were randomly grouped. Needle-and-thread suture (4–0 polypropylene, Johnson & Johnson) and the HFEW method are used for side-to-side anastomosis of the cecum in New Zealand rabbits (Fig. 1A and B). The sutures used for manual suturing were absorbable sutures commonly used in surgery. HFEW equipment consists of two components: a pulse generator and the anastomotic tool. Homemade pulse generator was developed to provide square-wave pulses to the intestinal tissue, operating at 440 kHz frequency with a voltage of 60 V (Fig. 1B). The anastomosis took 5 seconds with a 75% duty cycle.

### In vivo side-to-side anastomosis

The surgery was performed by professional surgeons of Huashan Hospital. Manual suturing was done with interrupted sutures (Fig. 1E–J), and HFEW anastomosis was done with a linear anastomosis made in-house in this study (Fig. 1K–N). For details regarding pre-operative preparations, intraoperative procedures, and post-operative care, please refer to the supporting information (Supplemental Digital Content SI, Methods, available at: http://links.lww.com/JS9/G555).

### Surgical parameters

The surgical time for intestinal anastomosis was defined as the total duration required to either weld or hand suture the bowel, which was measured using a stopwatch. During the procedure, bleeding was evaluated using the dry gauze absorption method. An infrared thermal imager (Fortric-200) was utilized to continuously monitor the temperature at the anastomosis site throughout the experiment. This temperature indicated the highest reading recorded at the anastomosis.

### Vital signs assessment

On postoperative days 3, 5, 7, 14, 21, and 28, vital signs of the experimental animals in both the manual suturing group and the HFEW group were observed (Fig. 1C). The observed indicators included food intake, body weight, fecal output, and mental state scores (Supplemental Digital Content SI Methods and Supplemental Digital Content Table S2, available at: http://links.lww.com/JS9/G555).

### Anastomosis strength test

Bursting pressure (BP) is the highest pressure recorded by the pressure gauge during infusion^[25]^. BP testing characterizes the strength of the intestinal tissue, with higher BP implying greater compressive strength and a higher ability to resist tissue rupture at the anastomosis. The BP measurement setup consists of three main components: a peristaltic pump, a pressure sensor, and the intestinal samples to be tested (Supplemental Digital Content Figure S5, available at: http://links.lww.com/JS9/G555). The Control group’s BP test used normal tissue that had not been anastomosed.

### Pathological evaluation

According to the experimental arrangement, a certain number of New Zealand rabbits were euthanized and disposed of at 7, 14, 21, and 28 days after surgery. After opening the abdominal cavity, the cecum of New Zealand rabbits was identified, carefully observed, and documented. The tissue at the anastomosis was carefully cleaned and immediately placed in formalin solution. Sections were made along the central part of the AS for histologic evaluation by an experienced pathologist.

### Immunofluorescence

In order to study the healing characteristics and the inflammatory response of the AS, We meticulously performed immunofluorescence analysis employing antibodies targeting CD68, CD163, INOS, Arg-1, Ki67, CD31, MPO, and BFGF to identify the presence of macrophages, vascular endothelial cells, and fibroblasts in the anastomotic tissue.

### Statistical analysis

Data analysis was performed using Prism 8, presenting results as mean values ± SEM. Intra-group variances were evaluated through ANOVA. All statistical analyses were two-tailed, with significance set at *P* < 0.05. Corresponding figures depict statistical test results (ns, not significant; *P < 0.05, **P < 0.01, ***P < 0.001, ****P < 0.0001) as appropriate.

## Results

### Temperature induces collagen denaturation at the AS

**Figure 2.**
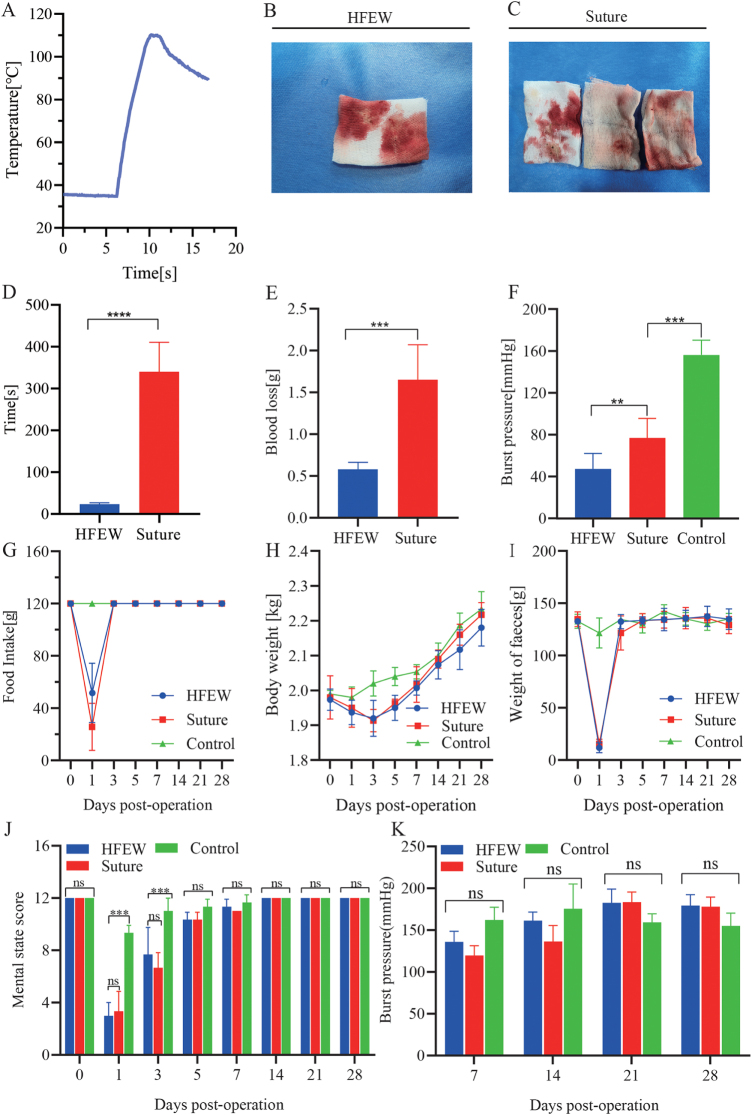
Experimental results of intraoperative and postoperative related indexes. (A) Changes of the highest anastomotic temperature in the HFEW group. (B and C) Bleeding in the HFEW combined Suture group. (D) Surgical elapsed time. (E) Surgical blood loss. (F) Burst pressure on the day of surgery. (G) Feed intake. (H) Weight change. (I) Fecal excretion. (J) Mental status score. (K) Postoperative burst pressure.

Temperature changes during anastomosis are shown in Figure 2A. The pulse generator discharged for a precise duration of 5 seconds, initiating the AS at an initial temperature of 35.4 °C, ultimately reaching a zenith of 109.8 °C (Fig. 2A; Supplemental Digital Content Figure S1, available at: http://links.lww.com/JS9/G555). Notably, the temperature surpassed the critical threshold of 70 °C within the very first 2 seconds. Drawing from pertinent antecedent research, it is well-established that tissue temperatures exceeding 70°C prompt collagen protein denaturation^[19]^. Due to the increase in temperature, tissues at the anastomotic site adhere instantly due to collagen thermal deformation, while this area also incurs thermal damage^[26]^.

### Surgical time consumption and bleeding

The average time consumed in the HFEW group was 23.6 ± 3.1 s, and the average time consumed in the Suture group was 339.8 ± 70.8 s, and the time consumed in the Suture group was significantly higher than that of the HFEW group (*P* < 0.05; Fig. 2D), and more than 10 times as long as that of the HFEW group. During the surgery, due to the thermal effect produced by the high-frequency current passing through the tissues, the temperature of the electrode contact site increased sharply, with the maximum temperature exceeding 100 °C (Supplemental Digital Content Figure S1, available at: http://links.lww.com/JS9/G555), and the temperature led to a certain degree of strength in the site, which also had a hemostatic effect. The average bleeding volume in the HFEW group was 0.58 ± 0.09 g, while in the Suture group, the average bleeding volume was 1.65 ± 0.42 g (Fig. 2B, C, and E). The bleeding volume in the Suture group was significantly higher than that in the HFEW group (*P* < 0.05) (Fig. 2E). Due to the shortening of the operation time, which was accompanied by a rise in tissue temperature during the operation, the outer surface of the tissue at the anastomosis in the HFEW group was flat and smooth, with almost no blood stains remaining (Fig. 1M and N). On the contrary, the outer surface of the tissue at the anastomosis in the Suture group showed a jagged shape with more blood stains remaining (Fig. 1H–J).

### Resume diet, body weight, bowel movements and mental state score

On postoperative day 1, both the HFEW and Suture groups showed significantly reduced food intake compared to the Control group (*P* < 0.05; Fig. 2G), with the HFEW group at 50% and the Suture group at 30% of baseline levels. By day 3, intake in both groups returned to levels comparable to the Control (*P* > 0.05; Supplemental Digital Content Figures S2 and S3, available at: http://links.lww.com/JS9/G555). Body weight in the HFEW and Suture groups reached their lowest point on postoperative day 3 but recovered to preoperative levels by day 5 (Fig. 2H; Supplemental Digital Content Figure S2, available at: http://links.lww.com/JS9/G555). Similarly, fecal output in both groups dropped to its nadir on day 1 post-surgery and returned to baseline by day 3 (Fig. 2I; Supplemental Digital Content Figure S2, available at: http://links.lww.com/JS9/G555). Mental state scores exhibited synchronized trends with dietary intake, body weight, and fecal output. Postoperative day 1 showed significantly lower mental state scores in the HFEW and Suture groups compared to Controls (*P* < 0.05). Scores began recovering by day 3, with HFEW group scores matching Controls by day 5 (Fig. 2J; Supplemental Digital Content Figure S4, available at: http://links.lww.com/JS9/G555).

### The strength of the AS increases gradually over time

At the end of the surgery, burst pressure testing of the tissue at the anastomosis was immediately performed (Supplemental Digital Content Figure S5, available at: http://links.lww.com/JS9/G555), and the results showed that the mean burst pressure was 163.00 ± 8.77 mm Hg in the Control group, 47.25 ± 14.76 mm Hg in the HFEW group, and 76.82 ± 18.67 mm Hg in the Suture group (Fig. 2F). Before healing, the mean burst pressure was lower in the HFEW and Suture groups than in the Control group, and the mean burst pressure was lower in the HFEW group than in the Suture group (*P* < 0.05).

As the wound healing time increased, the burst pressure gradually increased. On the seventh postoperative day, the values of burst pressure in the HFEW and Suture groups were 135.70 ± 12.92 mm Hg and 119.63 ± 11.68 mm Hg, respectively, and there was no significant difference between the two groups (*P* < 0.05; Fig. 2K), which indicated that the surgical site had already possessed the initial strength on the seventh postoperative day.

### Postoperative changes in AS external morphology

**Figure 3.**
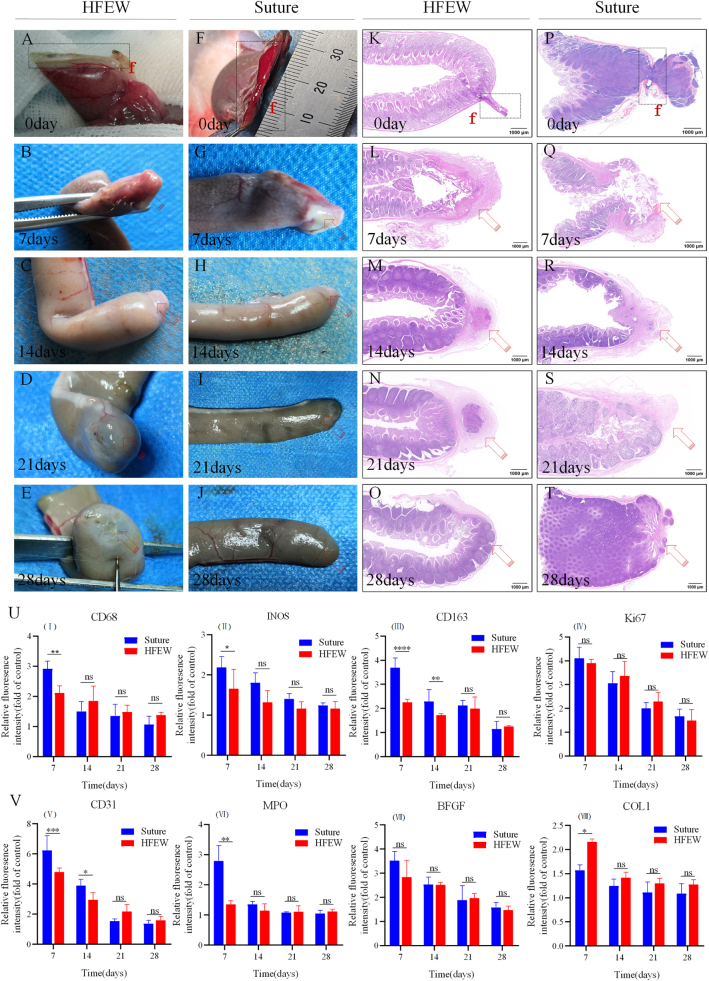
Macrostructural changes of intestinal tissues after surgery. (A–J) Macrostructural characteristics of intestinal tissues in the HFEW group and Suture group. (K–T) Micropathological structural characteristics of intestinal tissues in the HFEW and Suture group. The dashed box shows the tissue organization at the anastomosis. “f” refers to the fusion area, and the arrowheads refer to the tissue at the anastomosis that is healing. (U and V) Relative fluorescence intensity of CD68(I), INOS(II), CD163(III), Ki67(IV), CD31(V), MPO(VI), BFGF(VII), and COL1(VIII) antibodies compared to the control group. Bar graphs depict mean values ± SEM.

Normal cecum tissue has a smooth outer surface and a pouch-like end (Supplemental Digital Content Figure S6, available at: http://links.lww.com/JS9/G555). At the immediate end of surgery, the tissue at the anastomosis in the HFEW group protruded outward, with tightly joined ends and no foreign body residue (Fig. 3A); the tissue at the anastomosis in the Suture group had blood stains remaining and the tissue at the anastomosis was loosely joined (Fig. 3F). On the seventh postoperative day, the necrotic tissue protruding outward disappeared in the HFEW group (Fig. 3B); blood stains disappeared in the Suture group, and the tissue at the anastomosis was blurred (Fig. 3G). On the fourteenth postoperative day, complete connections were formed at the end of the cecum in the HFEW and Suture groups (Fig. 3C and H). On 21 and 28 post operation, the end remodeling was further improved in the HFEW and Suture groups, with angiogenesis on the surface (Fig. 3D, E, I, and J).

### Histopathological analyses of the AS

In the control group, the intestinal structure displayed clear layers, including the mucosal layer, submucosal layer, muscularis propria, and serosa (Supplemental Digital Content Figure S7, available at: http://links.lww.com/JS9/G555). In the HFEW group, the dissected tissues were continuously anastomosed with necrotic outwardly protruding dense tissue at the anastomosis, which was compressed to form a bilayer structure (Fig. 3K; Supplemental Digital Content Figure S8, available at: http://links.lww.com/JS9/G555). In the Suture group, the dissected tissue was anastomosed intermittently through the suture, and the anastomosis formed a “trumpet-shaped” irregular double-layer structure (Fig. 3P; Supplemental Digital Content Figure S8, available at: http://links.lww.com/JS9/G555). On day 7 postoperatively, necrotic tissue disappeared in the HFEW group, and granulation tissue filled the anastomosis (Fig. 3L; Supplemental Digital Content Figure S10, available at: http://links.lww.com/JS9/G555); in the Suture group, there was granulation tissue filling the anastomosis, but the structure was relatively loose, with more inflammatory cells (Fig. 3Q; Supplemental Digital Content Figure S10, available at: http://links.lww.com/JS9/G555). At day 14 postoperatively, the regenerative histopathologic structure at the anastomosis in the HFEW and Suture groups was further intact, with scarring composition (Fig. 3M and R; Supplemental Digital Content Figure S9 and S10, available at: http://links.lww.com/JS9/G555); and on day 21 postoperatively, the scar tissue at the anastomosis in the HFEW and Suture groups became smaller (Fig. 3N; Supplemental Digital Content Figure 3S, S9, and S10, available at: http://links.lww.com/JS9/G555). At day 28 after surgery, tissue remodeling was basically completed in the HFEW group and the Suture group, and the histopathological structure was basically restored to normal (Fig. 3O and T; Supplemental Digital Content Figure S9 and S10, available at: http://links.lww.com/JS9/G555).

### Immunofluorescence analyses of the AS

Based on the immunofluorescence intensity data, we analyzed different cytokines (Fig. 3U and V; Supplemental Digital Content Figure S11 and S12, available at: http://links.lww.com/JS9/G555). Macrophages (MPs) play a crucial role in wound healing and tissue regeneration processes^[27]^. To investigate the role of MPs in the reconstruction of the anastomosis site, we selected CD68 (total MPs), INOS (M1 marker), CD163 (M2 marker) as our study targets. M1-type MPs are pro-inflammatory macrophages, while M2-type MPs are anti-inflammatory macrophages^[28,29]^. CD68 and INOS peaked on day 7, at which time the relative values of CD68 and INOS in the Suture group were significantly higher than those in the HFEW group (2.91 vs 2.11; *P* < 0.05), and the difference between the two was not significant after 14 days. The relative content of CD163 in the HFEW group was significantly higher than that of the Suture group on days 7 and 14 (3.60 vs 1.20; *P* < 0.05). The results indicated that the inflammatory response was more intense in the Suture group, and the inflammatory response began to decline after 2 weeks.

Ki67 intertwines with cell proliferation^[30,31]^. CD31 is usually found in vascular endothelial cells and is used to assess angiogenesis^[32]^. The Ki67 content of HFEW and Suture groups reached its peak at day 7, and gradually decreased with the prolongation of healing time, and there was no significant difference in the Ki67 content between HFEW and Suture groups at the same time period (4.11 vs 3.37; *P* > 0.05). The CD31 content of the Suture group was significantly higher than that of the HFEW group at days 7 and 14 (6.23 vs 4.79; *P* < 0.05). This means that the tissue growth at the surgical site was faster in the Suture group on days 7 and 14.

Myeloperoxidase (MPO) is a crucial component of the innate immune system, primarily released by neutrophils to defend against invading pathogens^[33,34]^. At the seventh day, the MPO content was higher in the Suture group than in the HFEW group, and then there was no significant difference between them. The basic fibroblast growth factor (BFGF) stands out as a potent factor in promoting cell growth, which contribute to expediting the healing process of chronic ulcerative wounds^[35,36]^. The BFGF content of the HFEW and Suture groups peaked on the seventh day, but there was no significant difference between them. The collagen type I content of the HFEW group was higher than that of the Suture group on the seventh day, and it was not significant at other time periods.

### Wound healing stage of the AS

The results of vital signs characteristics, anastomotic strength, and pathological structural showed that the 7th and 14th postoperative days were critical for healing. On the 7th postoperative day, vital signs and tissue strength returned to normal, inflammatory reaction was intense, necrotic tissue disappeared, and granulation tissue regenerated. On day 14 postoperatively, the tissue inflammatory response diminished, scarring occurred, and vascular and epithelial tissue and collagen fibers generated. On day 28 postoperatively, scar deposition was reduced and tissue remodeling was essentially complete.

**Figure 4.**
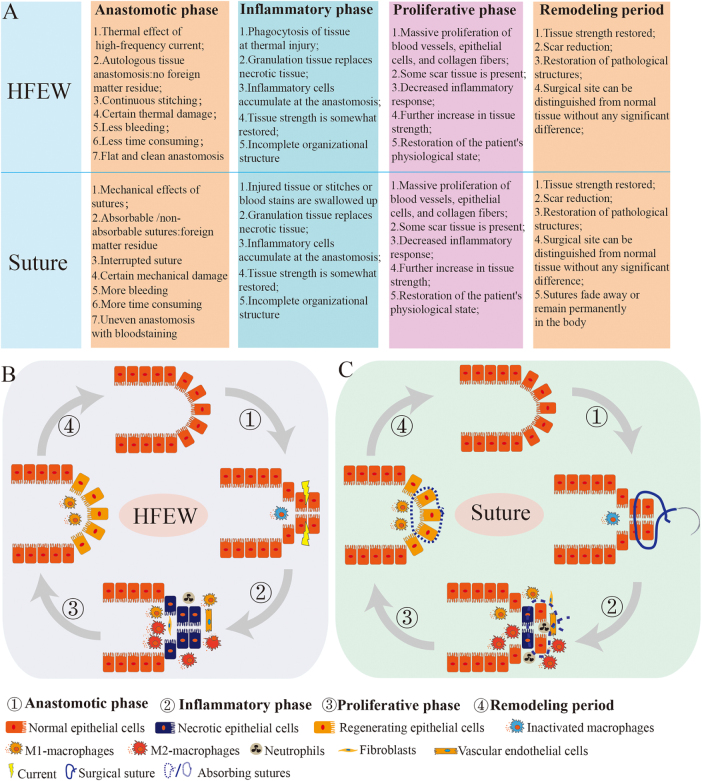
Schematic diagram of the surgical healing process of intestinal tissue in the HFEW and Suture groups. (A) Characterization of the four recovery phases in the HFEW and Suture groups. (B and C) Schematic diagram of surgical healing in the HFEW group and Suture group. Surgical healing all consisted of four phases, namely anastomotic phase, inflammatory phase, proliferative phase, and remodeling period.

Both anastomoses share a common postoperative recovery cycle, which includes the anastomotic, inflammatory, proliferative, and remodeling phases. The similarities, differences, and characteristics of the various healing stages are illustrated in Figure 4A–C. In comparison to the proliferative and remodeling phases, the variability exists mainly in the anastomotic and inflammatory phases (Fig. 4A). Inflammatory phase is the key to recovery, during which macrophages in the HFEW group phagocytose necrotic tissues and regenerative tissues appear. For the Suture group, due to the residual suture and blood stains, macrophages phagocytose the blood stains and residual sutures, and the inflammatory response is more intense. The proliferation and remodeling phases in the Suture group were similar to those in the HFEW group, with the residual sutures gradually disappearing over time, leading to a pathological structure that increasingly resembles normal tissue. In conclusion, the use of the HFEW method for cecum closure can effectively minimize the inflammatory response and expedite the surgical process, demonstrating a certain level of safety and reliability.

## Discussion

Conventional suturing is plagued by excessive bleeding and protracted operation times, while stapler anastomosis may result in the presence of residual foreign bodies. HFEW has the potential to circumvent these inherent drawbacks. Historically, research pertaining to the welding of intestinal tissue has predominantly been confined to *in vitro* investigations^[15,17,26]^, with limited reports of HFEW’s application in *in vivo* studies. Therefore, the present study focuses on comparing the physiological changes exhibited by animals after intestinal anastomosis using the HFEW and Suture technique, as well as the subsequent regenerative and repair processes that occur at the AS.

When using HFEW for soft tissue coaptation, the high-frequency electric current generates two effects through the tissue. The first is immediate adhesion caused by the thermal effect, and the second is tissue damage also induced by the thermal effect. Within the temperature range of 60–80 °C, tissue begins to whiten, and collagen undergoes denaturation^[37]^. Due to this, immediate adhesions are created in the tissues being welded, and a physical temporary connection is formed at the electrode contact site, along with some thermal damage to the tissues at the anastomosis. Due to the temperature, the risk of postoperative bleeding induced by the HFEW-based anastomosis technique is lower than that of needle and thread suture, and this is the reason why the amount of postoperative bleeding in the HFEW group in this study was one-third of that in the Suture group. Another major advantage of HFEW is the continuous anastomosis, which is made possible by the linear electrodes, and the results showed that the anastomosis time was only one-tenth of that of the Suture in the HFEW group. In fact, as an electrosurgical instrument, the HFEW does not leave any foreign body in the intestinal tissue, a feature that will allow more applicable scenarios for this technique in the future.

In the New Zealand rabbit cecal closure surgery, HFEW has a high level of security and reliability. This is mainly reflected in the following aspects. Firstly, the two surgical methods have the same survival rate, which is 100%. Secondly, the postoperative vital signs have the same trend of change, which decreased in a short period of time but quickly returned to normal. Thirdly, the postoperative changes in the strength of the tissue at the anastomosis are synchronous, and there is no anastomotic fistula or gastrointestinal bleeding during the recovery process. Fourth, the pathology results show that, 1 week after surgery, new tissue was generated, and 2 weeks after surgery, the tissue at the anastomosis began to remodel and the structure was the same as normal tissue. Last but not the least, the pathological results showed that 1 week after the operation, new tissue generated, and 2 weeks after the operation, the tissue at the anastomosis began to remodel, and the structure was closer to the normal tissue. While no adverse events were observed, the small sample size and limited follow-up duration suggest these findings should be interpreted cautiously. Longer-term studies with larger cohorts are warranted to confirm these preliminary results.

However, HFEW and Suture still have differences in the healing process. At the macroscopic level, this was mainly shown by the presence of thermal damage in the HFEW technique and the presence of foreign body residues in the intestinal tissues of the Suture group, unclean tissue at the anastomosis, blood stains and spilled intra-intestinal material, so that more risks existed in Suture group. Immunofluorescence results showed that the Suture group had more M1-type macrophages and neutrophils than the HFEW group, and therefore a more intense inflammatory response. Although the inflammatory response was more intense in the Suture group, the healing cycle was similar with that of the HFEW group, with an inflammatory phase, proliferative phase, and remodeling phase occurring sequentially after anastomosis. The inflammatory phase is the key to the healing of these two anastomoses, with the Suture group absorbing the absorbable suture and forming new granulation tissue, and the HFEW group absorbing the necrotic tissue and forming new granulation tissue. Based on the above analysis, HFEW can trigger a milder inflammatory response and contribute to wound healing. The healing and regeneration of intestinal tissues facilitated by HFEW consistently exhibit a gradual and slow pace, similar with the natural healing process of common wounds^[38,39]^.

Based on the experimental results of this study, it is necessary to believe that HFEW is a surgical anastomosis method with high research value, and its unique properties enable it to have a wide range of applications. Future surgical procedures applying HFEW include, but are not limited to, lateral–lateral anastomosis, end-to-end anastomosis, and end-to-side anastomosis. Surgical procedures using HFEW for anastomosis will also be expanded to include vascular, small bowel, colon, and gastric. However, it has to be recognized that the development of HFEW technology is currently at a preliminary level. In this paper, a preliminary comparative study of HFEW and Suture was performed without clinical studies in large animals, and the results have some limitations. When conducting safety and reliability studies, this study was conducted only on needle and thread sutures and was not compared with stapler anastomoses, which is another limitation of this study. Needle-and-thread suturing remains the surgical gold standard as the oldest technique. HFEW demonstrates superior blood loss reduction compared to traditional suturing. Both staplers and conventional sutures leave permanent foreign bodies in tissue. These findings don’t affect our study’s validity or HFEW’s clinical potential. Future research will develop an HFEW-based side-to-side anastomosis device for large animal intestinal surgery, with comparative *in vivo* studies evaluating HFEW against sutures and staplers for safety and reliability. When performing *in vivo* experiments, the method adopted in this study was open surgery, which is more harmful to the experimental animals and is not conducive to wound healing, and the instruments need to be improved in the future to meet the requirements of laparoscopic surgery. Furthermore, future research should enhance statistical processing; we need to incorporate formal power calculations in future studies to pre-determine sample sizes.

## Conclusions

HFEW demonstrates three key advantages: (1) it establishes sufficient welding strength at anastomosis sites, ensuring fundamental safety; (2) the necrotic tissue at the weld line undergoes progressive healing, transitioning from temporary closure to permanent connection; (3) in New Zealand rabbit cecum closures, HFEW matches sutures in safety and reliability. Compared to suturing, HFEW’s side-to-side cecal anastomosis significantly reduces postoperative bleeding, shortens operative time, and decreases inflammatory responses. These distinctive advantages position HFEW as a potentially innovative technology for emergency surgical applications.

## Data Availability

The data that support the findings of this study are available from the corresponding author upon reasonable request.
